# The additive effect of IgE-mediated and pseudoallergic hypersensitivity in RBL-2H3 cells and guinea pigs

**DOI:** 10.1371/journal.pone.0340855

**Published:** 2026-02-05

**Authors:** Yu Zhang, Qilong Xu, Chengbo Zheng, Cunyu Li, Yunfeng Zheng, Guoping Peng

**Affiliations:** 1 Affiliated Hospital of Nanjing University of Chinese Medicine, Jiangsu Province Hospital of Chinese Medicine, Nanjing, Jiangsu, China; 2 School of Pharmacy, Nanjing University of Chinese Medicine, Nanjing, Jiangsu, China; 3 National Key Laboratory on Technologies for Chinese Medicine Pharmaceutical Process Control and Intelligent Manufacture, Nanjing, Jiangsu, China; Yantai Institute of Technology, CHINA

## Abstract

Drug hypersensitivity reactions (DHR) are commonly observed with various medications. However, some drugs, like Qingkailing (QKL), iodixanol, and vancomycin, previously classified as pseudoallergic, do not align with clinical observations, creating uncertainty in understanding DHR mechanisms and implementing prevention. In response, our research team hypothesized that IgE-mediated allergy and pseudoallergic hypersensitivity may synergistically lead to more severe reactions. We established an IgE-mediated hypersensitivity model using an anti-dinitrophenyl IgE monoclonal antibody in RBL-2H3 cells and employed ovalbumin (OVA) for guinea pigs. Compound 48/80 served as a positive control for non-immunologic hypersensitivity. We measured β-hexosaminidase and histamine released in cells and guinea pigs to focus on hypersensitivity responses from both IgE-mediated and pseudoallergic pathways. Results indicated that combining pseudoallergic drugs and IgE significantly increased the hypersensitivity response compared to IgE or separate compound 48/80 groups. This effect was mirrored in the OVA-induced model. Notably, the three pseudoallergic drugs exhibited a similar increase in IgE-mediated hypersensitivity. These findings highlight the additive effects of IgE-mediated and pseudoallergic hypersensitivity, suggesting a more potent DHR response. The study also measured several cytokines and developed a method to differentiate between allergic, pseudoallergic DHRs, and additive reactions, aiding in understanding and controlling serious DHR risks in clinical practice.

## Introduction

Drug hypersensitivity reactions (DHRs) impose a substantial clinical burden—implicated in roughly one-third of adverse drug reactions and affecting 10–20% of hospitalized patients and up to 7% of the general population [1]. Yet a century of study has not tamed their unpredictability: for many drugs, mechanisms remain unsettled, and idiosyncratic events still lack reliable strategies for prediction or prevention. Closing this gap will require sharper mechanistic insight and clinically actionable markers to anticipate, avert, and better control DHRs.

Current understanding of DHR mechanisms is continually evolving, yet challenges persist in achieving a consensus on definitions and clinical diagnostic criteria. Discrepancies in terminology across the literature further complicate the interpretation of DHRs [2–6]. The main mechanisms of DHRs include IgE-mediated DHRs, the pharmacological interaction with immune receptors (the p-i concept), the mast-related G protein-coupled receptor X2 (MRGPRX2) pathway, the activation of the complement system, and other mechanisms. In IgE-mediated DHRs, the drug act as a hapten, binding covalently to proteins and generating a new antigen. Subsequently, this antigen stimulates the high-affinity IgE receptor (FcεRI) and a series of downstream phosphorylation reactions, ultimately leading to an allergic response [5,7,8]. The p-i concept postulates that some drugs directly bind to immune receptor proteins (human leukocyte antigens, HLA, and T cell receptors, TCR) and that this interaction leads to a T cell-mediated hypersensitivity [9]. Some drugs could directly stimulate MRGPRX2, leading to the activation of the phospholipase C (PLC) pathway [10]. Some drugs can break down complement proteins to produce C3a and C5a, which stimulate C3aR and C5aR on mast cells, triggering DHRs [11,12]. Other mechanisms include the G protein-coupled receptor E2 (ADGRE2, EMR2) pathway [3,13], non-steroidal anti-inflammatory drugs (NSAIDs)-induced DHRs [14], and others.

However, these current principles are insufficient to fully explain certain DHR cases in clinical practice. Here are several drugs mentioned in this article as examples: Qingkailing (QKL), iodixanol, and vancomycin (Van) injections. QKL injection, as one of the traditional Chinese medicine injections, has caused numerous DHRs. Initially, studies of DHRs caused by QKL injection consisted mainly of clinical case reports. From 1995 to 2008, there were more than 400 published case reports showing that QKL injection caused DHRs [15]. The symptoms of DHRs induced by QKL injection are mainly characterized by high fever, decreased blood pressure, asthenia, dyspnoea, nausea, vomiting, anaphylaxis, and, in severe cases, death [16,17]. Most DHRs are immediate, with most patients having an attack within 10–30 minutes and resolving within hours after stopping the drug [18]. Subsequently, an increasing number of cell and animal studies have investigated the DHRs induced by QKL injection. Studies have shown that it fails to induce IgE-mediated DHRs [19]. QKL injection could directly stimulate mast cells and basophils through C3a, leading to degranulation [19,20]. In some clinical cases, however, elevated total IgE levels were detected in patients’ serum [21,22]. These inconsistencies seem to suggest that we need to reconsider the mechanisms of DHR in clinical practice. For iodixanol, the onset time does not align with the commonly accepted IgE-mediated immediate drug allergy reactions; at the same time, some serum samples from certain cases exhibited characteristics of IgE-mediated DHR [23–25]. Van is generally considered to cause nonallergic DHR by the MRGPRX2 pathway [26,27]. However, 10% of DHR case reports suggest a possible IgE-mediated mechanism based on clinical presentations, positive skin testing results, and breakthrough symptoms despite premedication or during desensitization [28].

Clinical observations indicate that some pseudoallergy-inducing drugs present with features resembling IgE-mediated reactions. We therefore propose an additive model of DHR: in patients with pre-existing IgE-mediated sensitization, subsequent pseudoallergic stimulation from the injection amplifies mediator release and precipitates severe reactions.

In this study, we utilized IgE monoclonal antibodies to establish an RBL-2H3 cell model for IgE-mediated hypersensitivity and employed ovalbumin (OVA) to create a guinea pig model of IgE-mediated hypersensitivity. Additionally, we utilized compound 48/80 as a positive control for pseudoallergic hypersensitivity. Furthermore, iodixanol, Van, and QKL injections served as pseudoallergic drugs. Our experiments evaluated the degranulation rate of RBL-2H3 cells and measured the levels of released β-hexosaminidase and histamine in the cells and the blood plasma of guinea pigs. The aim of this study was to investigate the potential overlap between IgE-mediated hypersensitivity and pseudoallergic DHR, and determine whether they could synergistically lead to additive hypersensitivity.

## Methods

### Reagents

Dulbecco’s modified Eagle medium (DMEM), penicillin, and streptomycin were obtained from KeyGEN BioTECH, Nanjing, China. Fetal bovine serum (FBS) was obtained from Gibco, MA, USA. The p-nitrophenyl-N-acetyl-β-d-glucosaminide (NAG), histamine, o-phthalaldehyde (OPA), and Van hydrochloride were obtained from Shanghai Yuanye Bio-Technology Co., Ltd., Shanghai, China. Compound 48/80, OVA, and monoclonal anti-dinitrophenyl antibody (Anti-DNP IgE), produced in mice, were obtained from Sigma-Aldrich, Dorset, UK. Albumin from bovine serum 2,4-dinitrophenylated (DNP-BSA) was obtained from Invitrogen, Carlsbad CA, USA. Iodixanol Injection (100g:32g, Lot: 2015121) was obtained from Nanjing Chia Tai Tianqing Pharmaceutical Co., Ltd., Nanjing, China. QKL Injection (Lot:220416A2) was obtained from China Shineway Pharmaceutical Group Limited, Shijiazhuang, China.

### Cells and animals

Rat basophilic leukemia-2H3 (RBL-2H3) cells were obtained from the Chinese Academy of Sciences Cell Bank, Shanghai, China. The RBL-2H3 cell line was cultured in a complete growth medium composed of DMEM, 10% (v/v) FBS, 100 U/mL penicillin, and 100 μg/mL streptomycin. The cells were cultured in an incubator at 37°C, 5% CO_2_, and 95% humidified atmosphere. All animal experiments were performed in the experimental animal center of Nanjing University of Chinese Medicine based on the previous protocols [29,30]. These protocols were complied with the Jiangsu Province laboratory animal management measures and were approved by the experimental animal center of Nanjing University of Chinese Medicine. Specifically, eighty Hartley guinea pigs (male, 300 g ± 50 g) were obtained from Pizhou Dongfang Breeding Co., Ltd., Pizhou, China.Guinea pigs in the experimental group were sensitized by intraperitoneal injection of 0.2 mL ovalbumin solution (5 mg/mL, equivalent to 3.33 mg/kg body weight) on alternate days for three times. On day 21 after the first injection, animals were anesthetized with ketamine (66.67 mg/kg) and xylazine (6.67 mg/kg), prepared as aqueous solutions (200 mg/mL and 20 mg/mL, respectively), and administered intraperitoneally (0.1 mL). After anesthesia, 0.2 mL ovalbumin solution was administered intramuscularly, followed by 0.2 mL Qingkailing injection via the femoral vein to induce challenge. Thirty minutes after challenge, 1 mL of blood was collected from the carotid artery using EDTA-2K as anticoagulant. Animals were subsequently euthanized by exsanguination through carotid artery blood withdrawal under deep anesthesia. All procedures were performed under adequate anesthesia to minimize suffering, and animals were carefully monitored during the experiments. The doses of ovalbumin and Qingkailing injection were based on previous study [31], and the anesthesia regimen was chosen according to published report [32].

### IgE-mediated hypersensitivity cell and animal models

The anti-DNP IgE and DNP-BSA were used to induce IgE-mediated hypersensitivity in RBL-2H3 cells. According to the protocols [33–35], 2 × 10^5^ cells were cultured with 200 ng/ml anti-DNP IgE in a 48-wells plates for 24 hours; following this, 500 ng/ml DNP-BSA was added simultaneously with test drug to induce an IgE-mediated hypersensitivity. Additionally, OVA was used to induce IgE-mediated hypersensitivity in guinea pigs based on previously established methods [29,30]. Briefly, guinea pigs were intraperitoneally injected with 5 mg/kg of OVA saline solution on day 1, day 3 and day 5. On day 21, guinea pigs received an intramuscular injection of OVA at 5 mg/kg in saline, administered concomitantly with the study drug to induce an IgE-mediated hypersensitivity response.

### Morphological assay

The morphology of RBL-2H3 cells directly observed using a morphological method [36]. The 2 × 10^5^ cells were cultured in a 48-well plate for 24 hours. After drug treatment, the cell shape and granules observed through a Nikon ECLIOSE Ts2 microscope.

### MTT assay

Cells were taken and placed in a 96-well plate at 90 μL per well, then incubated overnight in a 37°C, 5% CO_2_ incubator. Drugs were added according to the experimental requirements. Then, 20 μL of MTT solution at a concentration of 5 mg/mL was added to each well, and incubation continued for 4 hours. The supernatant was discarded, and 150 μL of dimethyl sulfoxide (DMSO) solution was added to each well. The plate was shaken on an orbital shaker for 10 minutes to fully dissolve the crystals. The absorbance at 490 nm was measured using a microplate reader, and the cell viability of each group was calculated.

### β-hexosaminidase assay

The release of β‐hexosaminidase was assayed by a spectrophotometric method [37]. The culture supernate was collected and 200 μL of Tyrode solution with drugs were added into each well for 30 minutes. After drug stimulation, the supernatant was removed and the 200 μL of 0.1% TritonX-100 solution was added to lyse the cells for 30 min. A total of 50 μL of the supernatants and cell lysates were incubated with 0.1 mol/L of sodium citrate containing 0.001 mol/L of N-acetylglucosamine (NAG) (50 μL, pH 4.5) at 37°C for 2 hours. Finally, the carbonate buffer (200 μL, pH 10) composed of 0.1 mol/L Na2CO3 and 0.1 mol/L NaHCO3, which was used to terminate the reaction. The absorbance was measured at 405 nm. The absorbance of Tyrode solution with the corresponding drugs was used as blanks; the β-hexosaminidase assay release rate was calculated as following: β-hexosaminidase rate = Abs _supernatant_/ (Abs _supernatant_ + Abs _cell lysate_).

### Histamine assay

The release of histamine was measured by a fluoro spectrophotometry method [38]. The culture supernate was drawn and 200 μL of Tyrode solution containing the drugs was added to each well for 30 minutes. After drug stimulation, the supernatant was removed and 200 μL of 0.1% TritonX-100 solution was added to lyse the cells for 30 min. A total of 100 μL of the supernatant and cell lysates was mix with 0.5 mol/L NaOH (40 μL) and 2.5 g/L OPA (20 μL) and then incubated for 30 minutes. At the end of the incubation, 3 mol/L HCl (10 μL) was added. The fluorescence intensity was assayed by a fluorescent plate reader at 365 nm excitation wavelength and 465 nm emission wavelength. The fluorescence intensity of Tyrode solution with corresponding drugs was used as blanks. The histamine release rate was calculated as follows: Histamine release rate = F _supernatant_/ (F _supernatant_ + F _cell lysate_).

### ELISA

The histamine ELISA kit and guinea pig C3a ELISA kit were purchased from Shanghai Enzyme-linked Biotechnology Co., Ltd., Shanghai, China. The guinea pig IgE, IL-4, IL-13, C5a, SC5b-9, and IL-6 ELISA kits were purchased from Wuhan Fine Biotech Co., Ltd., Wuhan, China [39]. Blood plasma samples from guinea pigs were harvested in tubes containing heparin 30 minutes after drug administration and then centrifuged at 3000 rpm for 10 minutes. The plasma samples were separated. Following the instructions of the ELISA kits, the concentrations of the samples were assayed by a Tecan SPARK 10M plate reader.

### Statistical analysis

Data were analyzed by using GraphPad Prism Version 9.3.1 for Windows, (GraphPad Software, La Jolla, CA, USA). One-way analysis of variance (ANOVA) followed by Tukey’s test, Two-way ANOVA followed by Sidak’s test, and unpaired student’s t-test were used for comparing means. Results were considered significant with p-values smaller than 0.05.

### Ethics statement

This study was carried out in accordance with the principles of the Basel Declaration. All animal studies were approved by the Experimental Animal Ethics Committee, Nanjing University of Chinese Medicine (202206A061).

## Results

### The hypersensitivity responses of IgE-mediated allergy and compound 48/80 induced hypersensitivity in RBL-2H3 cells

**Fig 1:**
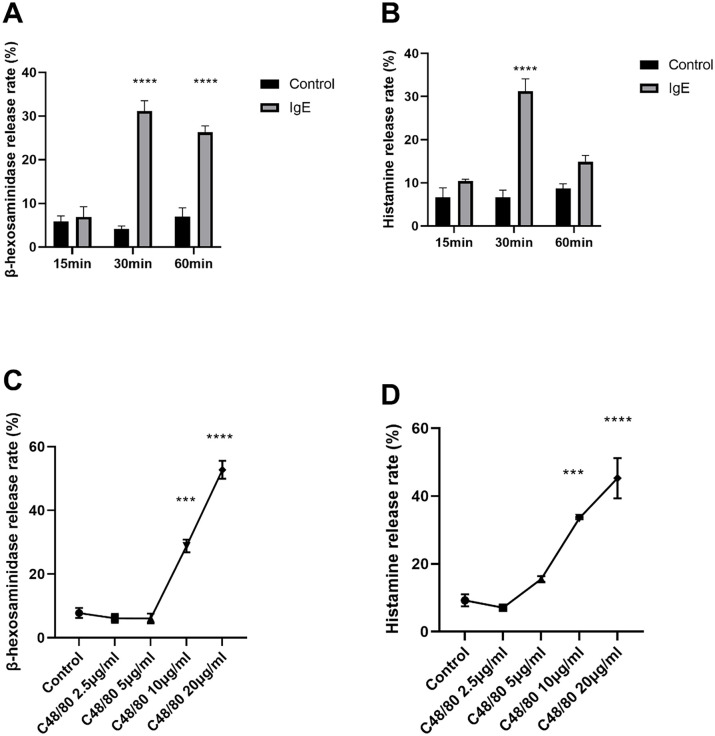
The β-hexosaminidase and histamine release rates of RBL-2H3 cells treated with 200 ng/ml anti-DNP IgE and 500 ng/mL DNP-BSA for from 15 minutes to 60 minutes and with 2.5 μg/mL-20 μg/mL compound 48/80 for 30 minutes. n = 3. Values indicate mean±SEM. Two-way ANOVAs followed by the Sidak test were employed compared to the control group. *, P < 0.05; **, P < 0.005; ***, P < 0.0005; ****, P < 0.00005 compared with the control group.

The entire study investigated the potential additive effects of IgE-mediated hypersensitivity and pseudoallergic hypersensitivity from two perspectives: cells and animals. In the cell experiments, the individual effects of positive control drugs on RBL-2H3 cell hypersensitivity reactions were first determined [29]. These experiments focused on the outcomes of the two types of drug hypersensitivity. Therefore, the direct products of basophils degranulation, β-hexosaminidase and histamine were determined. 200 ng/mL anti-DNP IgE and 500 ng/ml DNP-BSA were used to construct the IgE-mediated allegry cell model (**Fig 1A-1B**). The results showed that at both 30 min and 60 min, the rate of β-hexosaminidase release in the IgE group was 31.18 ± 1.35% versus 26.26 ± 0.87%, respectively, which was significantly higher compared to the control group. At 30 min, the rate of histamine release in the IgE group was 31.25 ± 2.86%, which was significantly higher than that of the control group. But at 60 min, the rate of histamine release decreased to 14.85 ± 1.5%, showing no significant difference, possibly due to the catabolism of histamine. Also, Svensson et al. found that histamine showed a rapid decline after 30 min in their cellular study of anesthetics [40]. Therefore, the duration of drug stimulation should be set at 30 minutes. Compound 48/80 was used as a pseudoallergic positive control drug. The RBL-2H3 cells were treated with a rage of 2.5–20 μg/mL compound 48/80 (**Fig 1C-1D**). The results showed that 10 and 20 μg/mL compound 48/80 increased both β-hexosaminidase and histamine levels. To verify the additive effect, the levels of both allergic and pseudoallergic hypersensitivity should not only be maintained at a level significantly different from the normal state but should also not fully trigger the release of hypersensitivity mediators from basophils. Therefore, 10 μg/mL of compound 48/80, which stimulates a “mild” hypersensitivity response, should be used to perform the next additive effect experiment.

### The additive effect of IgE-mediated hypersensitivity and compound 48/80 induced hypersensitivity in RBL-2H3 cells and guinea pigs.

**Fig 2:**
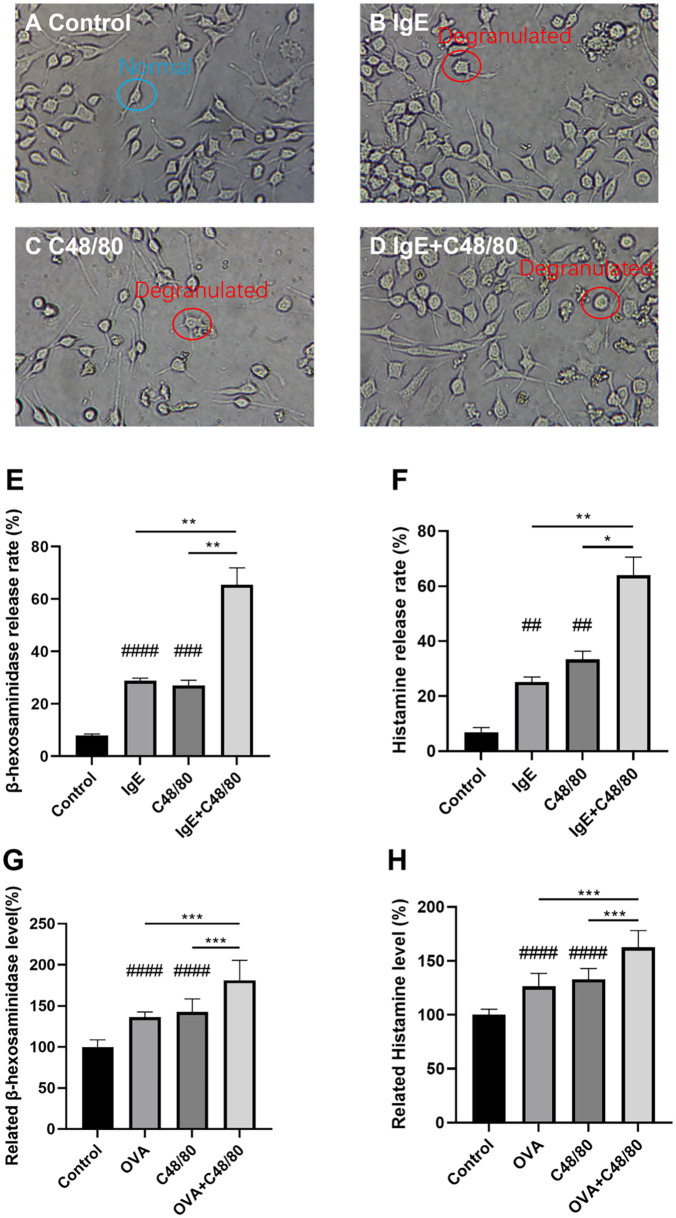
RBL-2H3 cells treated with H_2_O, 10μg/mL Compound 48/80. For IgE-positive groups, 200 ng/mL anti-DNP IgE was added and incubated for 24 hours, and then 500 ng/mL DNP-BSA were added at the same time as drug treatment. PBS was added to IgE-negative groups as vehicles of anti-DNP IgE and DNP-BSA. **A, B, C, D:** Cellular morphology of RBL-2H3 cells Magnification: × 200. **E, F:** β-hexosaminidase and histamine release rates of RBL-2H3 cells. n = 3. Values indicate mean±SEM. Unpaired student t-tests were employed compared to the control group. Eight guinea pigs per group were respectively injected into 0.9% NaCl, 0.75 mg/kg Compound 48/80. The 2 mg OVA was injected on Day 1, Day 3, and Day 5. On Day 21, another 2 mg OVA was injected to induce IgE-mediated allergy. **G, H:** Related β-hexosaminidase and histamine levels in guinea pigs. Values indicate mean±SD. Unpaired student t-tests were employed between each group. *#, P < 0.05; ##, P < 0.005; ###, P < 0.0005; ####, P < 0.00005,* compared to control group. *, P < 0.05; **, P < 0.005; ***, P < 0.0005; ****, P < 0.00005.

After determining the dose and timing for drug selection, the next step was to verify whether IgE-mediated hypersensitivity and compound 48/80-induced pseudoallergic hypersensitivity can coexist in RBL-2H3 cells and guinea pigs. For cell experiments, the degree of hypersensitivity was detected from two aspects: enzymatic reaction detection of β-hexosaminidase and fluorescence detection of histamine. Fig 2A-2D shows the morphology of RBL-2H3 cells treated with 10 μg/mL compound 48/80, anti-DNP IgE, and a combination of both. For examples, the normal RBL-2H3 cells in blue circle exhibit a slightly flattened morphology, with some cells elongating into spindle-shaped or irregular forms while the degranulated RBL-2H3 cells shows a round or oval shape. The morphological statistics are not precise; therefore, we performed β-hexosaminidase and histamines tests. The results indicated that the β-hexosaminidase and histamine release rates in the IgE + compound 48/80 group were 65.36% ± 6.35 and 64.02% ± 6.57, which were both significantly higher compared to both the allergy model group and the compound48/80 group. The pseudoallergic positive control drug compound 48/80 increased the hypersensitivity response from the allergy model cells and produced a more severe DHR and vice versa.

Moreover, after the confirmation of the additive effect of IgE-mediated and compound 48/80-induced hypersensitivity, the guinea pig hypersensitivity model was used for further validation *in vitro*. The OVA was used to induce IgE-mediated hypersensitivity [29,30]. The 0.75 mg/kg compound 48/80 was used to build pseudoallergic hypersensitivity [29,30]. The concentrations β-hexosaminidase and histamine in guinea pig blood plasma were tested to determine the hypersensitivity response. The results of related β-hexosaminidase levels compared to the control group were as follows: OVA: 136.19% ± 6.43; compound 48/80: 142.59% ± 15.76; compound 48/80 + OVA: 181.11% ± 24.33. Compared to the Control group, the OVA group increased by approximately 36%, while the compound 48/80 group increased by about 42%. The combined use of OVA and compound 48/80 resulted in an increase of 81% (**Fig 2G**). The histamine levels compared to the control group were as follows: OVA: 126.22% ± 12.14; compound 48/80: 132.89% ± 10.07; compound 48/80 + OVA: 162.57% ± 15.48. Compared to the Control group, the OVA group increased by approximately 26%, while the compound 48/80 group increased by about 32%. The combined use of OVA and compound 48/80 resulted in a 62% increase (**Fig 2H**). In guinea pigs, the 81% and 62% increases in the expression of β-hexosaminidase and histamine, respectively, after the combined use of OVA and compound 48/80 were both higher than the sum of the percentage increases observed with each of OVA or compound 48/80 used alone. These results align with the definition of an additive effect. Thus, we confirmed the presence of this additive effect both *in vivo* and *in vitro*.

### The hypersensitivity response of individual drugs in RBL-2H3 cells

After demonstrating the additive effects of IgE-mediated and compound 48/80-induced pseudoallergic hypersensitivity, we aimed to investigate whether the class of pseudoallergic drugs used in clinical settings could also exhibit additive effects with IgE-mediated hypersensitivity. As mentioned before, three pseudoallergic drugs were selected, namely, QKL injection, iodixanol, and Van. First, we determined the hypersensitivity reactions triggered by each individual drug in RBL-2H3 cells. Selection based on the study of the most appropriate concentrations for hypersensitivity reactions to individual drugs. For each candidate drug, we established four different concentrations for gradient experiments. Drug exposure was 30 min to match anti-DNP IgE and compound 48/80; β-hexosaminidase and histamine release quantified activation and guided dosing for subsequent experiments.

**Fig 3:**
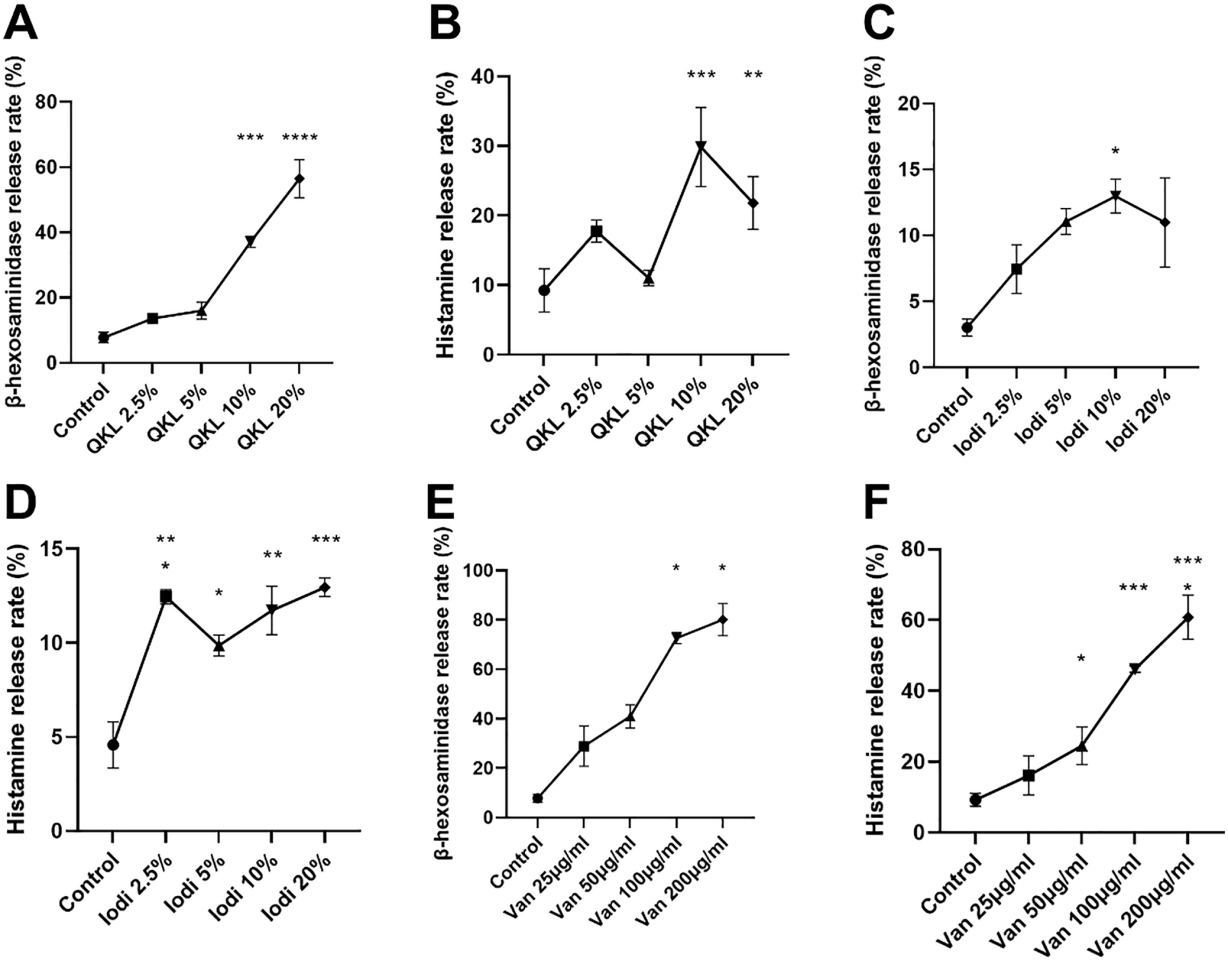
The β-hexosaminidase and histamine release rates of RBL-2H3 treated with 3 drugs. Cells were respectively treated with H2O, 10% QKL, 10% iodixanol, and 100 μg/ml vancomycin for 30 minutes. The 200 ng/ml anti-DNP IgE was added and incubated for 24 hours, and then 500ng/ml DNP-BSA were added at the same time as drug treatment. H_2_O was used as the vehicle of anti-DNP IgE and DNP-BSA. Qingkailing injection = QKL; iodixanol = Iodi; vancomycin = Van. n = 3.Values indicate mean±SEM. One-way ANOVA followed by Tukey test were employed. *, P < 0.05; **, P < 0.005; ***, P < 0.0005; ****, P < 0.00005.

The results showed that all three drugs significantly increased the secretion of β-hexosaminidase and histamine in RBL-2H3 at the two highest concentrations among the four concentrations tested. According to these results, 10% QKL injection, 10% iodixanol, and 100 μg/mL Van were used in the following experiments to verify the additive effect in RBL-2H3 cells (Fig 3A-3F).

Furthermore, to confirm that the observed hypersensitivity responses were not due to cytotoxic effects, cell viability was assessed under all drug treatment conditions. The MTT assay results demonstrated that none of the treatments, either alone or in combination with IgE, significantly affected RBL-2H3 cell viability (see S1 Fig).

**Fig 4:**
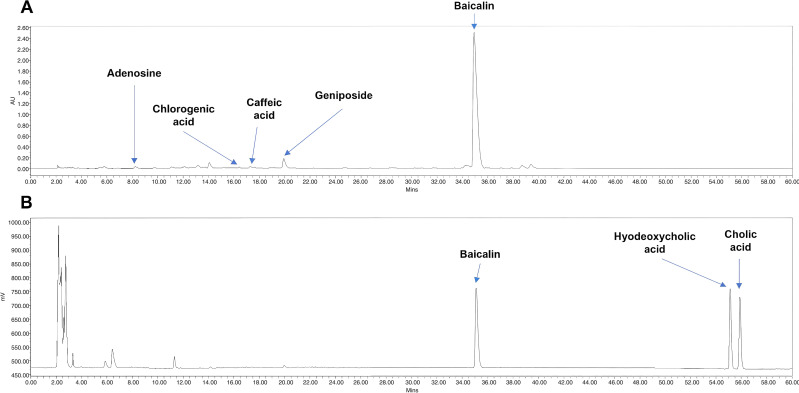
Determination of components of QKL by HPLC. **(A) UV absorption at 254 nm; (B) ELSD.** Column: Hypersil ODS-2 C18 column (4.6 mm × 250 mm, 5 μm). Mobile phases: methanol (A) and 0.1% formic acid in water **(B)**. The gradient was as follows: 0-5 min: 5% A, 95% B; 5-10 min: 25% A, 75%; 10-40 min: 60% A, 40% B; 40-50 min: 80% A, 20% B; 50-60 min: 90% A, 10% **B.** Solvent flow rate of 1 mL/min. Temperature: 35°C.

Besides, the components of QKL injection are highly complex, including cholic acid, porcine deoxycholic acid, and extracts of Isatidis radix, Lonicera japonica Thunb., Cape jasmine, nacre, and buffalo horn. Different brands of QKL may have slight variations of ingredients in their production processes. This study also utilized high-performance liquid chromatography (HPLC) to identify the active components in QKL injection (**Fig 4**). The concentration of the main components of QKL injection as follows: baicalin, 6.5353 mg/mL; cholic acid, 7.1264 mg/mL; hyodeoxycholic acid, 9.2325 mg/mL; geniposide, 0.3050 mg/mL; caffeic acid, 0.0159 mg/mL; chlorogenic acid, 0.0097 mg/mL; adenosine, 0.0170 mg/mL.

### The additive effect of IgE-mediated allergy and pseudoallergic hypersensitivity in RBL-2H3 cells and guinea pigs

After determining the extent of hypersensitivity reactions induced by the three drugs, we aimed to assess whether these three drugs could interact with IgE to produce an additive effect. The experiment was similar to previous additive effect experiments, where compound 48/80 was replaced with QKL injection, iodixanol injection, and Van, and the degranulation rate, β-hexosaminidase levels, and histamine levels were measured to determine whether these drugs could also exhibit an additive effect with anti-DNP IgE.

**Fig 5:**
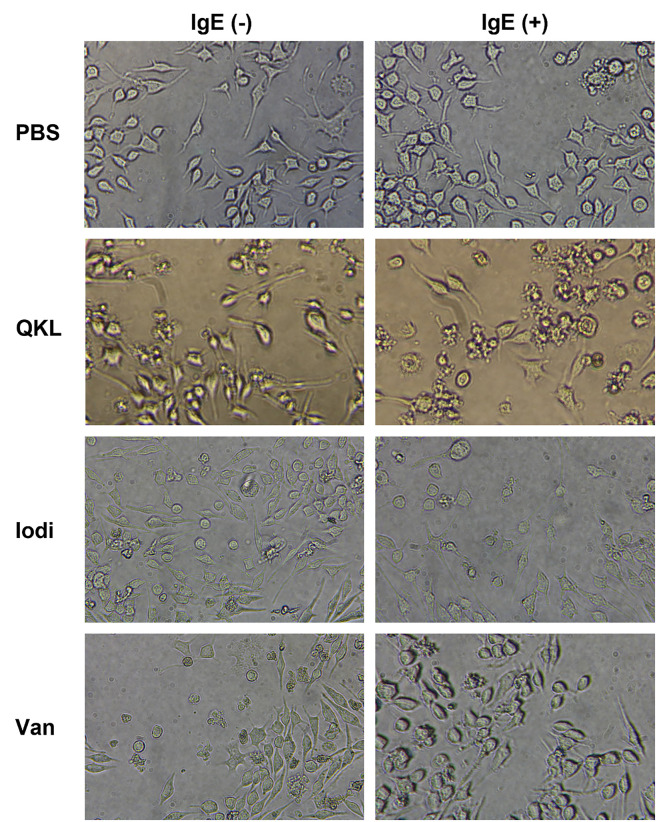
RBL-2H3 cells treated with H_2_O, 10μg/mL compound 48/80, 10% QKL, 10% iodixanol, and 100 μg/ml vancomycin for 30 minutes. For IgE-positive groups, 200 ng/ml anti-DNP IgE was added and incubated for 24 hours, and then 500 ng/ml DNP-BSA were added at the same time as drug treatment. PBS was added to IgE-negative groups as vehicles of anti-DNP IgE and DNP-BSA. Magnification: × 200.

**Fig 6:**
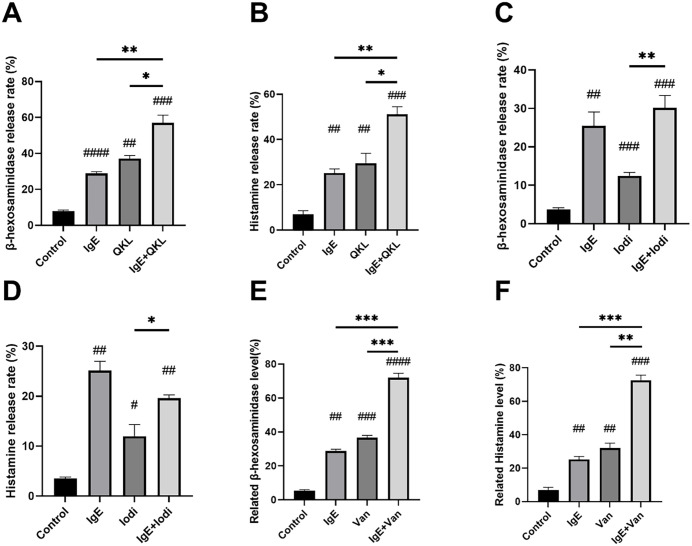
The β-hexosaminidase and histamine release rates of RBL-2H3 cells treated with 10% H_2_O, 10 μg/mL compound 48/80, 10% QKL, 10% iodixanol, and 100 μg/ml vancomycin. For IgE-positive groups, 200ng/ml anti-DNP IgE was added and incubated for 24 hours, and then 500ng/ml DNP-BSA were added at the same time as drug treatment. PBS was added to IgE-negative groups as vehicles of anti-DNP IgE and DNP-BSA. n = 3. Values indicate mean±SEM. Unpaired student t-tests were employed between each group. *, P < 0.05; **, P < 0.005; ***, P < 0.0005; ****, P < 0.00005 compared with the control group. *#, P < 0.05; ##, P < 0.005; ###, P < 0.0005; ####, P < 0.00005* compared with the control group.

**Fig 5** shows the morphology of RBL-2H3 cells. **Fig 6** shows the results of β-hexosaminidase and histamine release rates. The results indicate that QKL and Van combination group (anti-DNP IgE + pseudoallergic drug) exhibited a significant increase compared to the IgE group or the single drug group (**Fig 6A**, **6B**, **6E and 6F**). The β-hexosaminidase release rate in the stacked group was significantly higher compared to the iodixanol group, while no difference was observed in the allergy model (**Fig 6C**). The same was true for the histamine release rate assay (**Fig 6D**).

**Fig 7:**
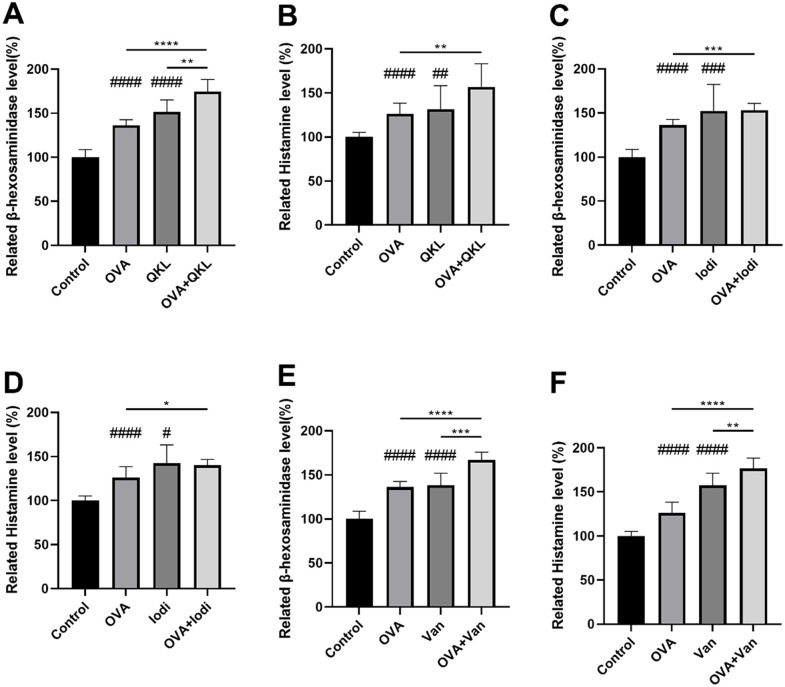
Related β-hexosaminidase and histamine levels in guinea pigs. Eight guinea pigs per group were respectively injected into 0.9% NaCl, 0.2 ml QKL, 0.2 mL iodixanol, and 0.1 g/kg vancomycin. The 2 mg OVA was injected on Day 1, Day 3, and Day 5. On Day 21, another 2 mg OVA was injected to induce IgE-mediated hypersensitivity. Values indicate mean±SD. Unpaired student t-tests were employed between each group. *#, P < 0.05; ##, P < 0.005; ###, P < 0.0005; ####, P < 0.00005*, compared to control group. *, P < 0.05; **, P < 0.005; ***, P < 0.0005; ****, P < 0.00005.

In addition, we conducted guinea pig experiments to determine whether this additive effect also exists *in vivo*. To find the most suitable drug concentration, we first established an IgE hypersensitivity model using OVA and employed the aforementioned administration methods. We referred to the optimal blood collection time from previous experiments. Under the condition that the time variable remained constant, we used three concentrations to measure the levels of β-hexosaminidase and histamine in guinea pig plasma (see S2 Fig). Based on the experimental results, QKL and iodixanol were administered at a concentration of 0.2 mL per 300 g, while Van was administered at a concentration of 0.1 g/kg for subsequent experiments. The β-hexosaminidase and histamine concentrations in guinea pig blood plasma were tested to determine the hypersensitivity response. The results of β-hexosaminidase levels compared to the control group (**Fig 7**) were as follows: OVA: 136.19% ± 6.43; QKL: 151.53% ± 13.62; QKL + OVA: 234.25% ± 28.28; iodixanol: 152.34% ± 30.08; iodixanol + OVA: 209.35% ± 35.60; Van: 138.42% ± 13.43; Van + OVA: 179.64% ± 20.06. The results of related histamine levels compared to the control group were as follows: OVA: 126.22% ± 12.14; QKL: 131.81% ± 26.52; QKL + OVA: 231.86% ± 32.25; iodixanol: 142.42% ± 20.89; iodixanol + OVA: 202.69% ± 18.30; Van: 157.55% ± 13.67; Van + OVA: 197.37% ± 21.46. We can find that, for both β-hexosaminidase and histamine levels, the increase of the OVA + QKL group is greater than the increase of the growth of the OVA group and QKL group. This phenomenon also happens with the drug iodixanol while Van does not show this ‘1 + 1>2’ phenomenon. Overall, these animal experiments also demonstrated that for both β-hexosaminidase level and histamine level, all dual drug groups exhibited a significant increase compared to allergic hypersensitivity models or single drug treatment.

### The plasma characteristic of allergy, pseudoallergic hypersensitivity and additive reactions

**Fig 8:**
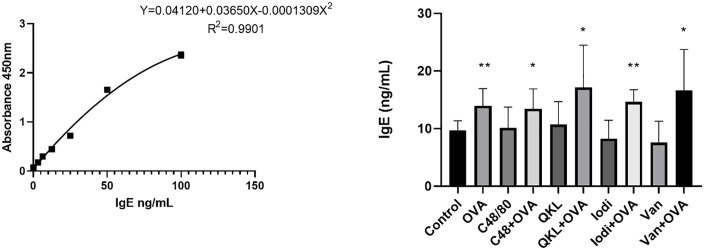
The concentration of IgE in the blood plasma of guinea pigs induced by OVA, compound 48, and QKL injection. Eight guinea pigs per group were treated with 0.9% NaCl (control), 0.75 mg/kg compound 48/80, 0.2 mL/300 g Qingkailing injection, 0.2 mL/300 g iodixanaol injection, and 0.1 g/kg vancomycin for 30 minutes. The 2 mg OVA was used following animal model method. The concentrations of IgE measured by Elisa kits. Values indicate mean±SD. Unpaired student t-tests were employed. *, P < 0.05; **, P < 0.005; ***, P < 0.0005; ****, P < 0.00005, compared to control group.

In addition to the β-hexosaminidase and histamine levels mentioned in the preceding paragraph, some cytokines were also measured. The concentrations of IgE in guinea pigs were measured by ELISA (**Fig 8**). The IgE level in the control group was 9.73 ± 1.64 ng/ml, whereas the level in the OVA group was 13.93 ± 2.99 ng/ml, which was significantly higher than that in the control group. There was no significant difference in the compound 48/80, QKL, Iodixanol, and Van groups, and all the combined groups were also significantly higher than the control group (For details, see S3**-**S6 Fig). This indicates that in additive hypersensitivity reactions, IgE levels are raised only due to allergic reactions.

**Fig 9:**
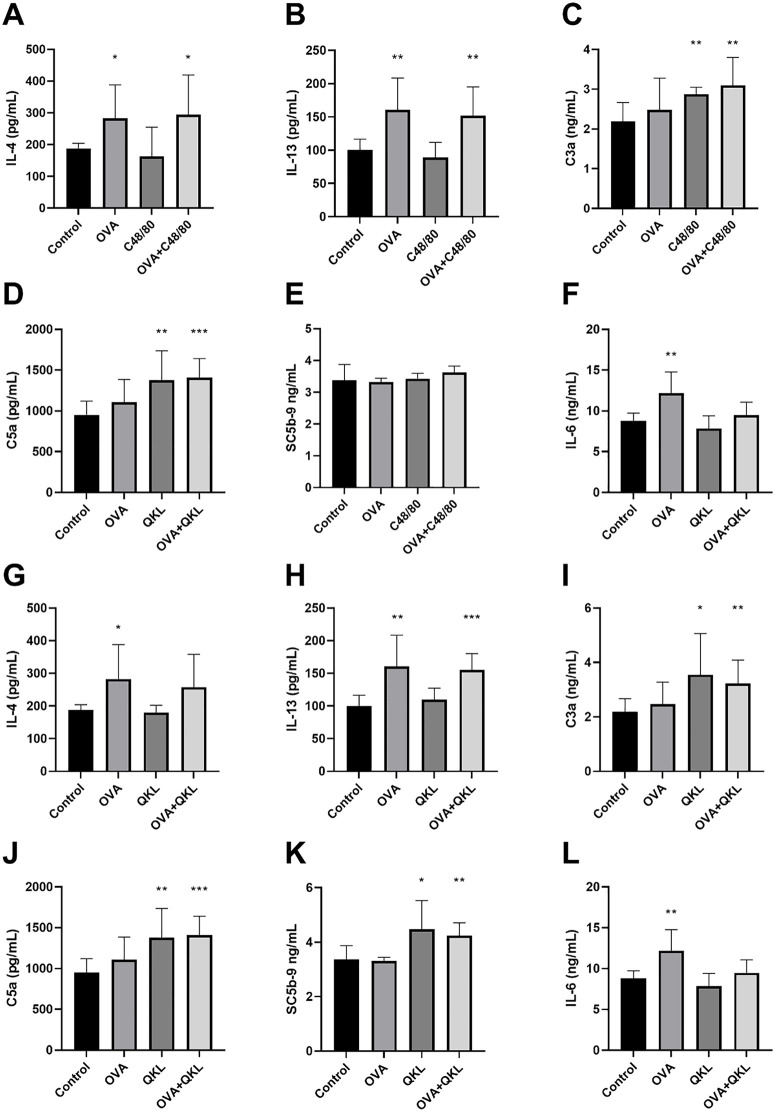
The concentration of cytokines in the blood plasma of guinea pigs induced by OVA, compound 48, and QKL injection. Eight guinea pigs per group were treated with 0.9% NaCl (control), 0.2 ml/300 g QKL injection, and 0.75 mg/kg compound 48/80 for 30 minutes. The 2 mg OVA was used following animal model method. The concentrations of IL-4, IL-13, C3a, C5a, SC5b-9, and IL-6 were measured by ELISA kits. Values indicate mean±SD. Unpaired student t-tests were employed. *, P < 0.05; **, P < 0.005; ***, P < 0.0005; ****, P < 0.00005, compared to control group.

**Fig 10:**
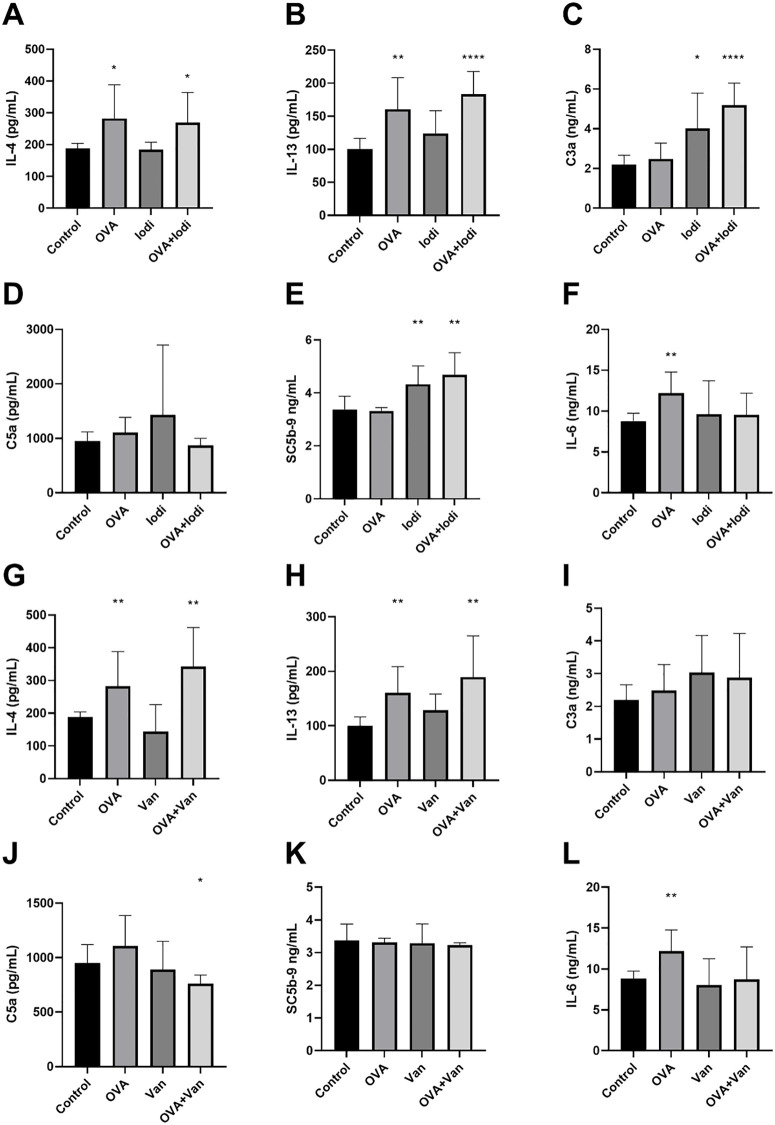
The concentration of cytokines in the blood plasma of guinea pigs induced by OVA, iodixanol, vancomycin and injection. Eight guinea pigs per group were treated with 0.9% NaCl (control), 0.2 ml/300 g iodixanol injection, and 0.1 g/kg vancomycin for 30 minutes. The 2 mg OVA was used following animal model method. The concentrations of IL-4, IL-13, C3a, C5a, SC5b-9, and IL-6 were measured by ELISA kits. Values indicate mean±SD. Unpaired student t-tests were employed. *, P < 0.05; **, P < 0.005; ***, P < 0.0005; ****, P < 0.00005, compared to control group.

The concentration of IL-4, IL-13, C3a, C5a, IL-6, and SC5b-9 in the blood plasma of guinea pigs were tested (**Fig 9 and Fig 10**). The results show that OVA could increase the IL-4, IL-13, and IL-6 levels (**Fig 9A**, **9B**, **9F**). The compound 48/80 increased the C3a and C5a levels (**Fig 9C**, **9D**). The QKL and iodixanol injection could stably increase the C3a and SC5b-9 levels (**Figs 9I**, **9K and 10C**, **10E**). Van did not increase any cytokines (Fig 10G-10L).

## Discussion

DHRs occur with many medications. However, current research does not fully explain some clinical cases, making it difficult to clarify mechanisms and prevent events. We propose a dual-mechanism hypothesis: in certain patients, DHRs may result from the combined or sequential activation of IgE-mediated and pseudoallergic pathways, leading to an amplified response. Using anti-DNP IgE and DNP–BSA, we established an IgE-mediated hypersensitivity model in RBL-2H3 cells, and used OVA to establish an IgE-mediated hypersensitivity model in guinea pigs. We first demonstrated that IgE-mediated DHRs and compound 48/80–induced pseudoallergic reactions produced additive responses in both RBL-2H3 cells and guinea pigs (**Fig 2**).

Subsequently, we verified that three drugs, QKL, iodixanol, and Van, also exhibited an additive effect with IgE-mediated allergic reactions in RBL-2H3 cells and guinea pigs. The stacked groups of QKL, iodixanol and Van showed significant differences compared to the IgE-mediated allergy as well as the individual drug *in vivo and in vitro* (**Figs 6 and 7**). For agents such as QKL and iodixanol, confirming an additive interaction between IgE-mediated allergy and pseudoallergic hypersensitivity may help explain challenging clinical cases. Some patients already have an allergic milieu (e.g., allergic rhinitis, RSV infection, parasitic infection) and have been sensitized through prior allergen exposure. In such patients, administration of a drug that triggers pseudoallergic reactions can precipitate a severe DHR. Severe DHRs may also occur when a pseudoallergic reaction coincides with an IgE-mediated reaction to another medication, or when additional allergenic components in the injection formulation (e.g., excipients) contribute additively.

To profile pathway-specific signals, we used IgE together with IL-4 and IL-13 as markers of IgE-mediated allergy [41,42], and C3a/C5a (via C3aR/C5aR) plus the terminal complement complex SC5b-9 as markers of complement-driven pseudoallergy; SC5b-9 is relatively stable and useful for detecting complement activation [43,44]. IL-6 showed a stable and significant increase in an allergy model in our previous study, so we also wanted to understand whether an additive effect occurs in the reactions. Based on the detection of IgE, IL-4, IL-13, C3a, C5a, SC5b-9, and IL-6 levels in guinea pig plasma, we found that IgE and IL-13 were indicators with a stable increase in the OVA allergy model, and that IL-4 and IL-6 could also be used as supportive indicators. For compound 48/80, QKL injection, and iodixanol injection, both C3a and SC5b-9 were indicators with a stable increase. IL-4 and C5a, although not showing a stable increase in all experiments, can also be used as supportive indicators. Moreover, the increased values of IL-4, IL-13, C3a, and C5a in the additive group were essentially equivalent to the increase produced by either the OVA allergy model or by drug stimulation alone, suggesting that the allergic and pseudoallergic indicators of the additive effect appear to be mutually exclusive. It is noteworthy that compound 48/80, as a pseudoallergic positive drug, also elevated C3a and C5a in guinea pigs, possibly revealing that its mechanism of action is not only the MRGPRX2 pathway, but also activates the complement pathway. Van, on the other hand, did not show significant elevations in IL-4, IL-13, C3a, C5a, SC5b-9, and IL-6, revealing that all of its immune stimuli were direct. In summary, in conjunction with the results of the β-hexosaminidase and histamine assays, we may be able to construct a method for distinguishing between IgE-mediated DHRs, pseudoallergic DHRs, and additive reactions. The β-hexosaminidase and histamine can be used as indicator of total drug hypersensitivity. The IgE and IL-13, aided by IL-4 and IL-6, are used to determine whether an allergic reaction has occurred. C3a and SC5b-9, aided by C5a, are used to determine whether a complement-activated anaphylactic-like reaction has occurred. If none of the other indicators increase, yet β-hexosaminidase and histamine rise and a drug hypersensitivity reaction (DHR) occurs, this pattern suggests direct, non-IgE-mediated activation of mast cells/basophils. In practice, these markers can be hard to capture: histamine is rapidly degraded and often undetectable, and C3a is quickly consumed *in vivo*. Some reports note that during DHRs the rate of C3a consumption may exceed its generation from C3 cleavage, further limiting its detectability.

The metabolite of C3a, C3a-desArg, can be used in clinical practice instead of C3a, which is produced through C3/C5 convertase generated by removing the C-terminal arginine of C3a. After conversion of C3a to C3a-desArg, it is less biologically active but more easily detectable [45]. IL-6 in our experiments can be used as an indicator, whereas in clinical practice the situation may be more complicated; patients may experience an inflammation, which can mislead judgement [46,47].

In conclusion, our study shows that IgE-mediated allergy and pseudoallergic hypersensitivity exhibit an additive effect, leading to a more serious DHR. We also constructed a method for distinguishing between allergy, pseudoallergic DHRs, and additive reactions. These works could help to understand and trace the reasons for the serious DHR incidents that have occurred. It also helps to control the risk of serious DHRs in clinical application.

## Supporting information

S1 Fig RBL-2H3 cells activity under different drug treatments. RBL-2H3 cells were divided into the following groups: purified water (Control), 200 ng/mL Anti-DNP IgE (IgE), 10 μg/mL Compound (C48/80), 10% Qingkailing Injection (QKL), 10% Iodixanol Injection (Iodi), and 100 μg/mL Vancomycin (Van). The cells were stimulated with the respective drugs for 30 minutes. In the addtive groups, 200 ng/mL Anti-DNP IgE was applied in addition to the respective drug treatments. The absorbance at 490 nm was measured. n = 5, data are presented as mean ± SD. Unpaired Student’ t-tests were performed, and compared with the control group, *, *P* < 0.05.
(TIF)

S2 Fig Histamine and β-hexosaminidase levels in guinea pig plasma. The contents of histamine and β-hexosidase were determined by ELISA. Among them, 0.9% NaCl (Control group), compoud 48/80 (compound 48/80 group), low (0.1 ml/300g), medium (0.2 ml/300g) and high (0.4 ml/300g) doses of Qingkiling injection (QKL L, M and H groups), low (0.05 ml/300g), medium (0. 1 ml/300g) and high (0. 2 ml/300g) doses of ioxanol injection (Iodi L, M and H groups), Low (50 mg/kg), medium (100 mg/kg) and high (200 mg/kg) doses of vancomycin (groups Van L, M and H). n = 5, the value represents the average ±SD. One-way ANOVA and Dunnett-t test were performed, *, P < 0.05; **, P < 0.005; ***, P < 0.0005; ****, P < 0.00005, compared to control group.
(TIF)

S3 Fig Levels of IL-4, IL-13, C3a, C5a, SC5b-9 and IL-6 determined by ELISA in plasma of guinea pigs stimulated by compound 48/80. *, P < 0.05; **, P < 0.005; ***, P < 0.0005; ****, P < 0.00005, compared to control group.
(TIF)

S4 Fig Levels of IL-4, IL-13, C3a, C5a, SC5b-9 and IL-6 determined by ELISA in plasma of guinea pigs stimulated by QKL injection. *, P < 0.05; **, P < 0.005; ***, P < 0.0005, compared to control group.
(TIF)

S5 Fig Levels of IL-4, IL-13, C3a, C5a, SC5b-9 and IL-6 in determined by ELISA plasma of guinea pigs stimulated by ioxanol injection. *, P < 0.05; **, P < 0.005; ****, P < 0.00005, compared to control group.
(TIF)

S6 Fig Levels of IL-4, IL-13, C3a, C5a, SC5b-9 and IL-6 in determined by ELISA plasma of guinea pigs stimulated by vancomycin. *, P < 0.05; **, P < 0.005, compared to control group.
(TIF)
