# Correction: The additive effect of IgE-mediated and pseudoallergic hypersensitivity in RBL-2H3 cells and guinea pigs

**DOI:** 10.1371/journal.pone.0351278

**Published:** 2026-06-08

**Authors:** Yu Zhang, Qilong Xu, Chengbo Zheng, Cunyu Li, Yunfeng Zheng, Guoping Peng

## Notice of Republication

An incorrect version of S1 Data was published in error. This article was republished on May 20, 2026 to correct this error. Please download this article again to view the correct version.
