# Supplementary figures and images for: The additive effect of IgE-mediated and pseudoallergic hypersensitivity in RBL-2H3 cells and guinea pigs
Source: PLoS One. 2026 Feb 5;21(2):e0340855. doi: 10.1371/journal.pone.0340855 (PMC13189007; doi:10.1371/journal.pone.0340855)

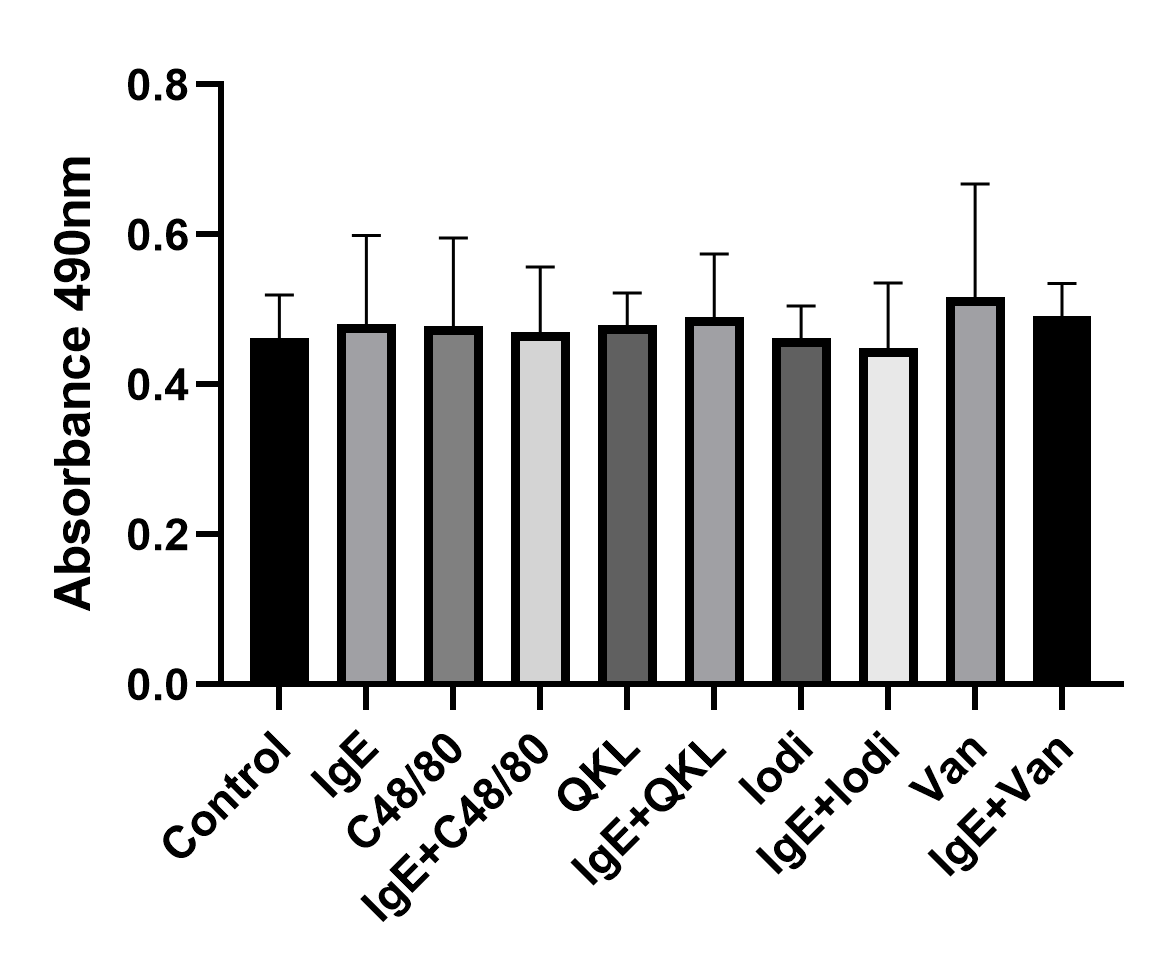

Supplement: S1 Fig — RBL-2H3 cells were divided into the following groups: purified water (Control), 200 ng/mL Anti-DNP IgE (IgE), 10 μg/mL Compound (C48/80), 10% Qingkailing Injection (QKL), 10% Iodixanol Injection (Iodi), and 100 μg/mL Vancomycin (Van). The cells were stimulated with the respective drugs for 30 minutes. In the addtive groups, 200 ng/mL Anti-DNP IgE was applied in addition to the respective drug treatments. The absorbance at 490 nm was measured. n = 5, data are presented as mean ± SD. Unpaired Student’ t-tests were performed, and compared with the control group, *, P < 0.05. (TIF) [file pone.0340855.s001.tif]

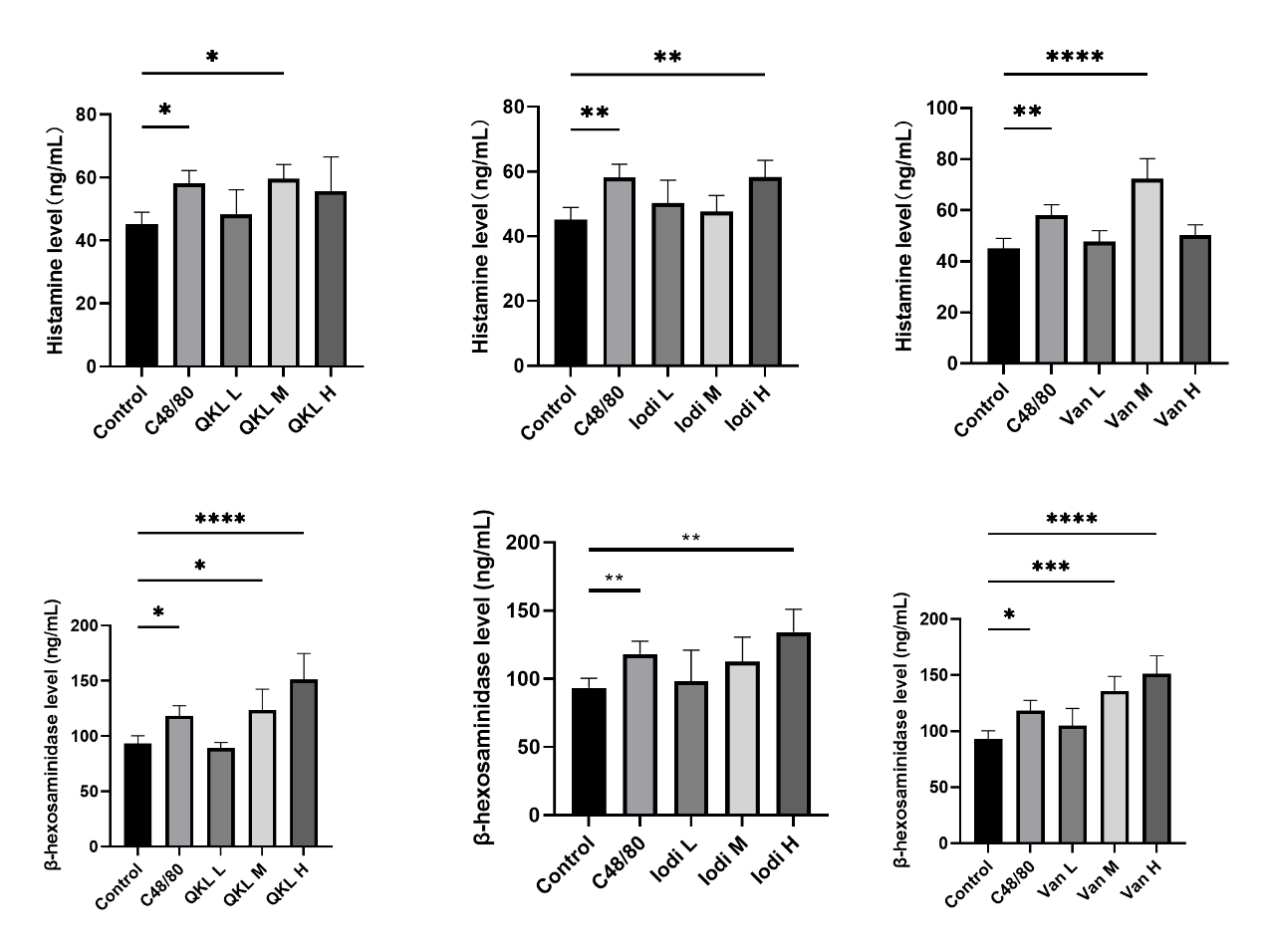

Supplement: S2 Fig — The contents of histamine and β-hexosidase were determined by ELISA. Among them, 0.9% NaCl (Control group), compoud 48/80 (compound 48/80 group), low (0.1 ml/300g), medium (0.2 ml/300g) and high (0.4 ml/300g) doses of Qingkiling injection (QKL L, M and H groups), low (0.05 ml/300g), medium (0. 1 ml/300g) and high (0. 2 ml/300g) doses of ioxanol injection (Iodi L, M and H groups), Low (50 mg/kg), medium (100 mg/kg) and high (200 mg/kg) doses of vancomycin (groups Van L, M and H). n = 5, the value represents the average ±SD. One-way ANOVA and Dunnett-t test were performed, *, P < 0.05; **, P < 0.005; ***, P < 0.0005; ****, P < 0.00005, compared to control group. (TIF) [file pone.0340855.s002.tif]

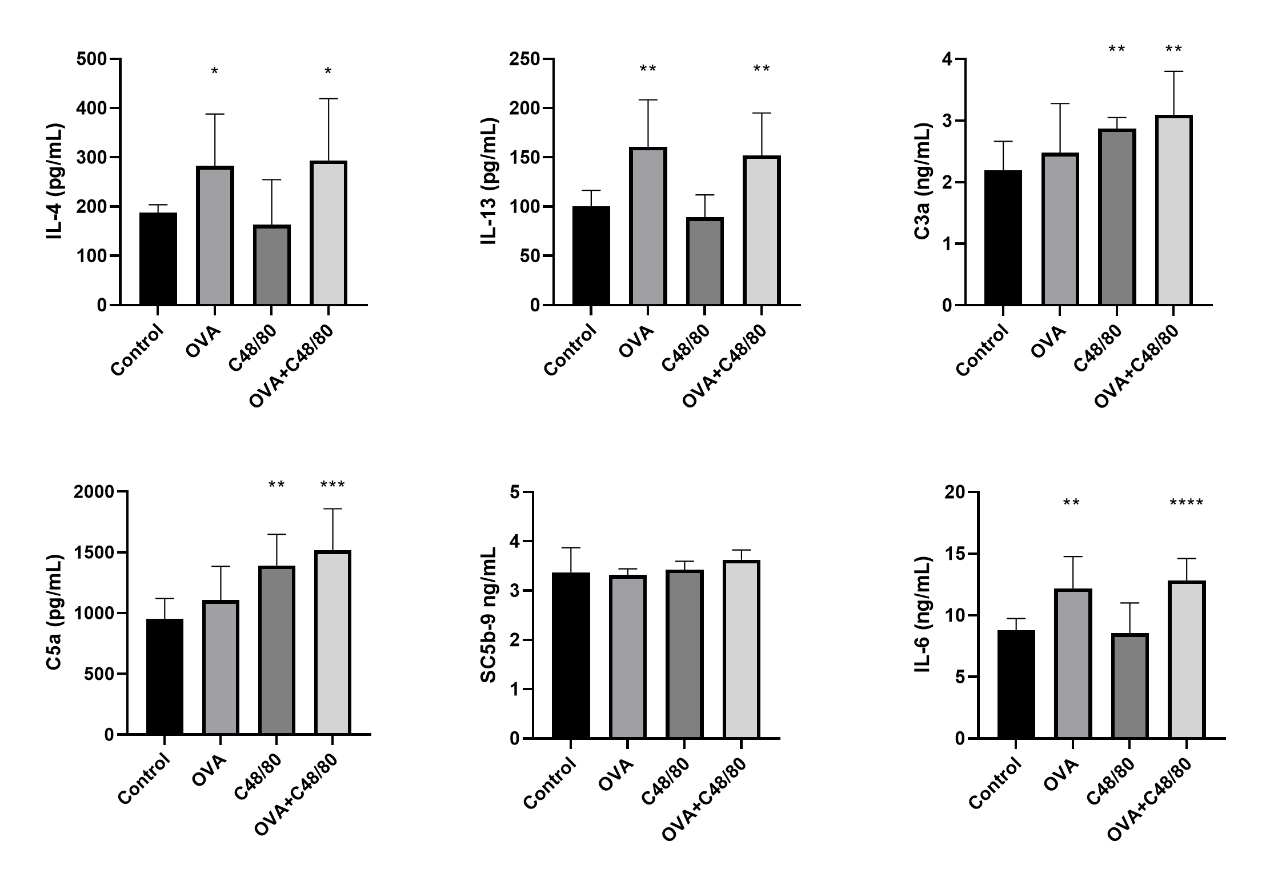

Supplement: S3 Fig — *, P < 0.05; **, P < 0.005; ***, P < 0.0005; ****, P < 0.00005, compared to control group. (TIF) [file pone.0340855.s003.tif]

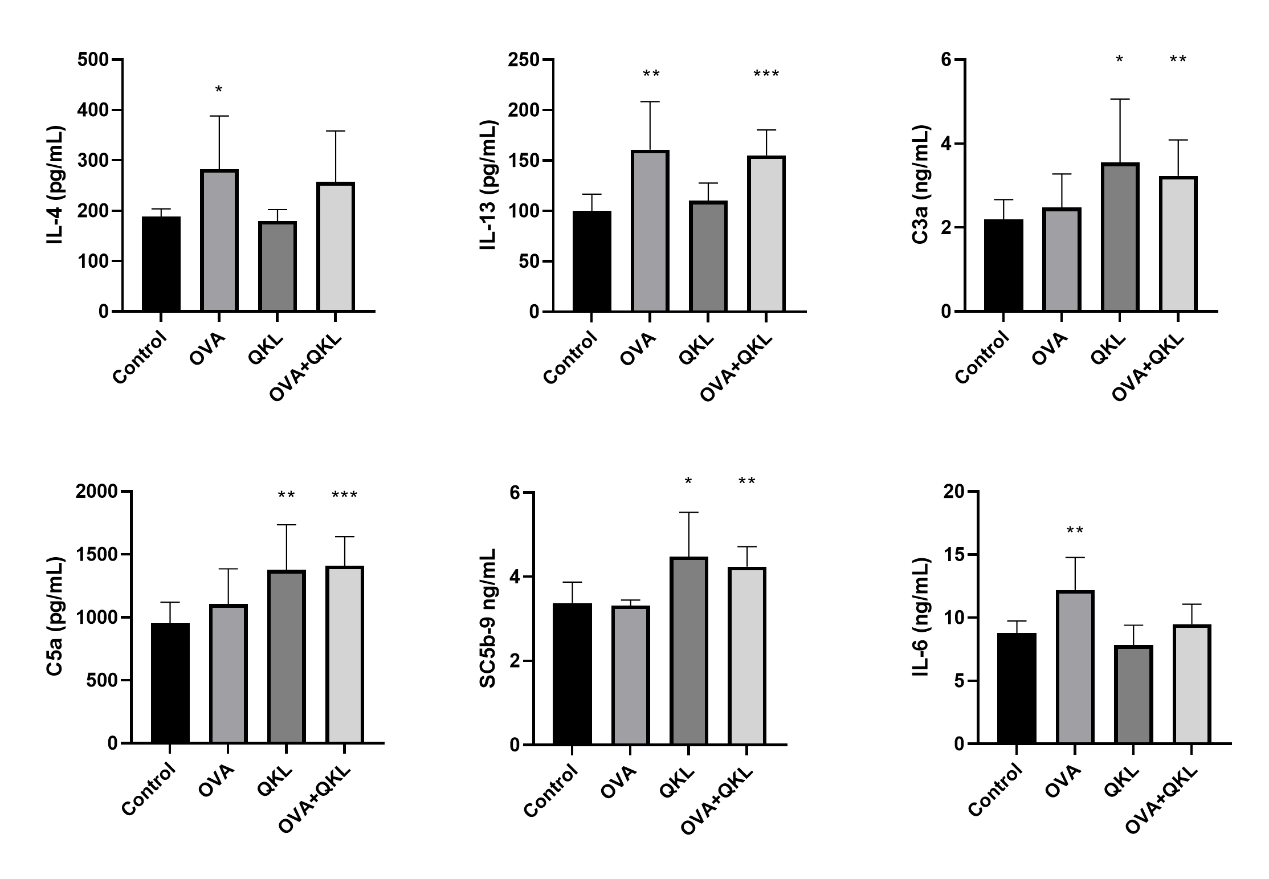

Supplement: S4 Fig — *, P < 0.05; **, P < 0.005; ***, P < 0.0005, compared to control group. (TIF) [file pone.0340855.s004.tif]

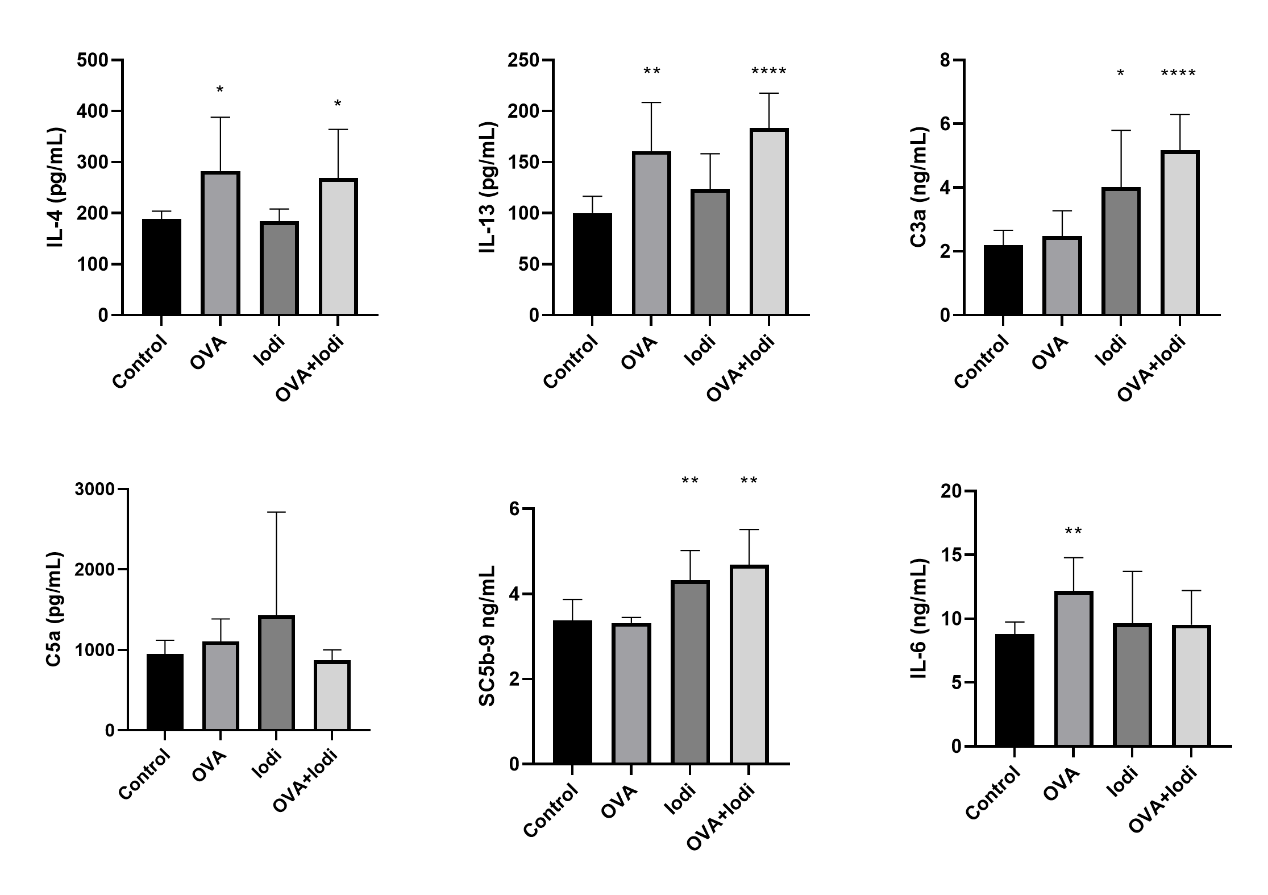

Supplement: S5 Fig — *, P < 0.05; **, P < 0.005; ****, P < 0.00005, compared to control group. (TIF) [file pone.0340855.s005.tif]

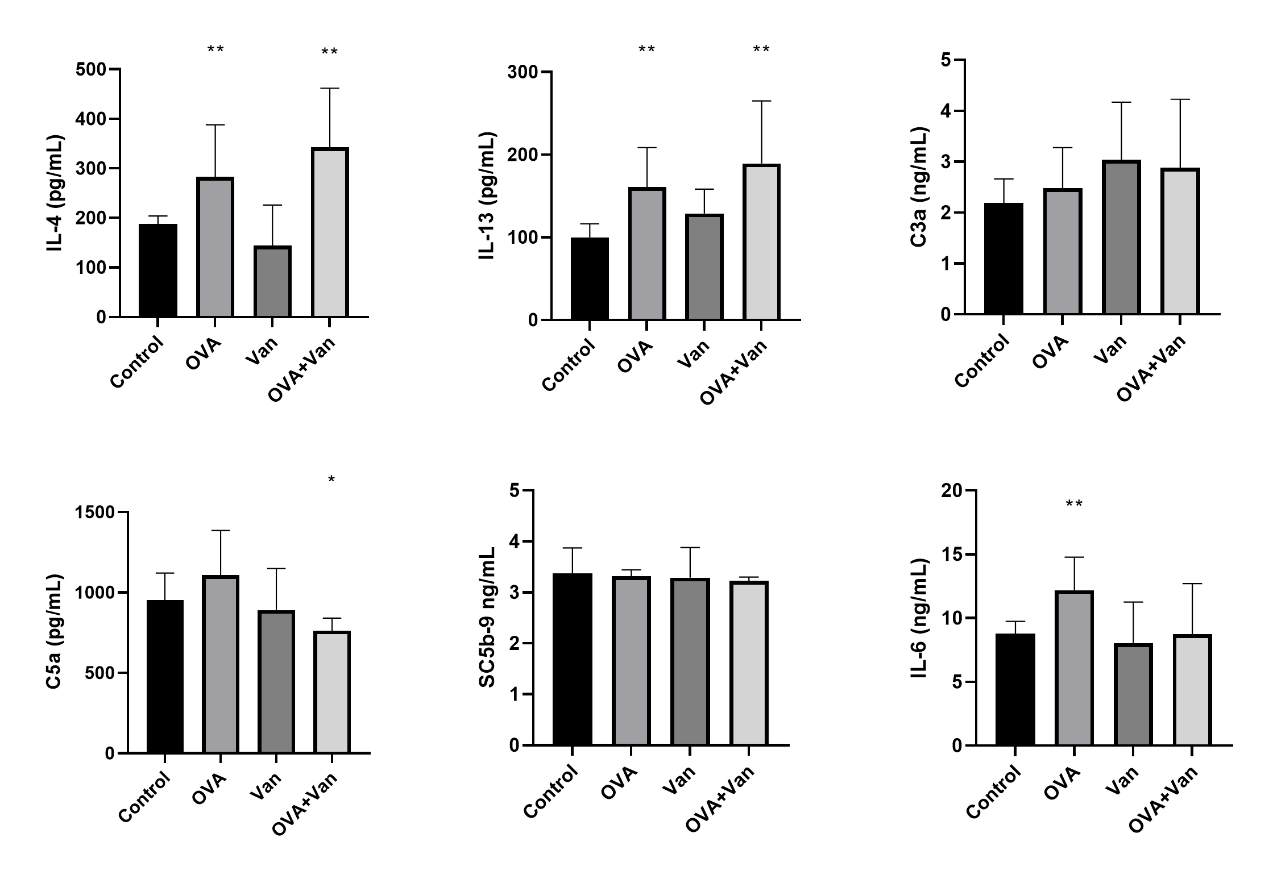

Supplement: S6 Fig — *, P < 0.05; **, P < 0.005, compared to control group. (TIF) [file pone.0340855.s006.tif]
